# Niche differentiation of comammox *Nitrospira* in sediments of the Three Gorges Reservoir typical tributaries, China

**DOI:** 10.1038/s41598-022-10948-9

**Published:** 2022-04-26

**Authors:** Jiahui Zhang, Mingming Hu, Yuchun Wang, Jianwei Zhao, Shanze Li, Yufei Bao, Jie Wen, Jinlong Hu, Mingzhi Zhou

**Affiliations:** 1 State Key Laboratory of Simulation and Regulation of Water Cycle in River Basin, Beijing, 10038 People’s Republic of China; 2 Department of Water Ecology and Environment, China Institute of Water Resources and Hydropower Research, Beijing, 100038 People’s Republic of China; 3 Laboratory of Eco-Environmental Engineering Research, State Environmental Protection Key Laboratory of Soil Health and Green Remediation, College of Resources and Environment, Huazhong Agricultural University, Wuhan, 430070 Hubei People’s Republic of China

**Keywords:** Microbiology, Biogeochemistry

## Abstract

Complete ammonia oxidizer (Comammox) can complete the whole nitrification process independently, whose niche differentiation is important guarantee for its survival and ecological function. This study investigated the niche differentiation of comammox *Nitrospira* in the sediments of three typical tributaries of the Three Gorges Reservoir (TGR). Clade A and clade B of comammox *Nitrospira* coexisted in all sampling sites simultaneously. The *amoA* gene abundance of clade A and B was gradually increased or decreased along the flow path of the three tributaries with obvious spatial differentiation. The *amoA* gene abundance of comammox *Nitrospira* clade A (6.36 × 10^3^ − 5.06 × 10^4^ copies g^−1^ dry sediment) was higher than that of clade B (6.26 × 10^2^ − 6.27 × 10^3^ copies g^−1^ dry sediment), and the clade A *amoA* gene abundance was one order of magnitude higher than that of AOA (7.24 × 10^2^ − 6.89 × 10^3^ copies g^−1^ dry sediment) and AOB (1.44 × 10^2^ − 1.46 × 10^3^ copies g^−1^ dry sediment). A significant positive correlation was observed between comammox *Nitrospira* clade A *amoA* gene abundance and flow distance (*P* < 0.05). The number of operational taxonomic units (OTUs) in two sub-clades of clade A accounted for the majority in different tributaries, indicating that clade A also had population differentiation among different tributaries. This study revealed that comammox *Nitrospira* in the sediments of TGR tributaries have niche differentiation and clade A.2 played a more crucial role in comammox *Nitrospira* community.

## Introduction

Nitrification transforms 2330 teragrams of nitrogen during the earth’s nitrogen cycle every year^1^. For over a century, nitrification process was considered to consist of two steps: first, ammonia was oxidized to nitrite, and then nitrite was oxidized to nitric acid^2,3^. However, the recent discovery of comammox has broken through traditional conception of two-step nitrification process^4,5^. As a bacterium associates with *Nitrospira* sublineage II, comammox can completely oxidize ammonia into nitrate in a single cell because it can encode all the enzymes required to complete nitrification^6,7^. Previous study has demonstrated that comammox *Nitrospira* can be classified into two clades, namely clade A and clade B, and clade A was further grouped into sub-clade A.1 and A.2^8^.

Many studies have revealed a wide existence of comammox *Nitrospira* in freshwater and terrestrial ecosystems such as basin^9^, lake^10^, riparian ecosystem^11^, coastal wetland^12^, agricultural soil^13^, and forest soil^14^. Comammox *Nitrospira* have also been found in engineering environments including drinking water systems^6,15^, wastewater treatment systems^16,17^ and activated sludge^18^. These studies show that comammox *Nitrospira* exhibit strong distinct ecological distribution. In some environments, *amoA* gene of comammox *Nitrospira* is undetectable^9^, but in others, comammox *Nitrospira* are the main ammonia-oxidizing microorganism (AOM). In addition, different lineages of comammox *Nitrospira* also show niche differences. Clade A.1 is the main lineage in mudflat sediments^19^, while clade A.2 and clade B are abundant in agricultural soils^20,21^. Clade A.1 is more widely distributed than clade A.2 in natural aquatic ecosystems^22^. Moreover, *Ca. Nitrospira inopinata*, belonging to clade A.1, is the only successfully cultured pure comammox *Nitrospira* strain^23^. Comammox *Nitrospira* clade B has a higher affinity for NH_4_^+^-N than clade A, which may be due to the existence of an ammonium transporter in clade B^7,24,25^. Such a niche differentiation ensures that comammox *Nitrospira* can widely survive in nature and exert their ammonia oxidation function.

The Three Gorges Reservoir (TGR), situated in the middle reaches of the Yangtze River of China, is the largest reservoir in the world with a length of 663 km, a water storage of 39.3 billion m^3^, and a water surface area of 1084 km^2^^26^. TGR has a function of a periodic anti-seasonal water storage and discharge, and it discharges water in the rainy season and stores water in the dry season every year with a water level of 145 − 175 m^27^. The storage capacity of the TGR tributaries accounts for about 25.0% of the total storage capacity of the reservoir area, and eutrophication has gradually appeared in TGR tributaries in recent years due to the enrichment of nutrients^28,29^. The main stream in the reservoir area exhibits a certain supporting effect on the tributaries in the process of integrating the tributaries into the main stream, which makes the flow velocity of the tributaries gradually slow down and the sediments gradually settle down, resulting in the spatial difference among the tributaries along the flow path.

One previous study on the Yangtze River continuum has shown that comammox *Nitrospira* in the sediments of Yangtze River mainstream present large-scale niche differentiation at different altitudes^30^. However, the distribution and diversity of comammox *Nitrospira* in the sediments of the TGR tributaries remain largely unknown. In this study, we hypothesized that the spatial differences among the TGR tributaries might lead to niche differentiation of comammox *Nitrospira* in the sediments. We investigated abundance, distribution, and diversity of comammox *Nitrospira* in sediments of three typical TGR tributaries, trying to test this hypothesis.

## Methods

### Study areas and sample collection

**Figure 1:**
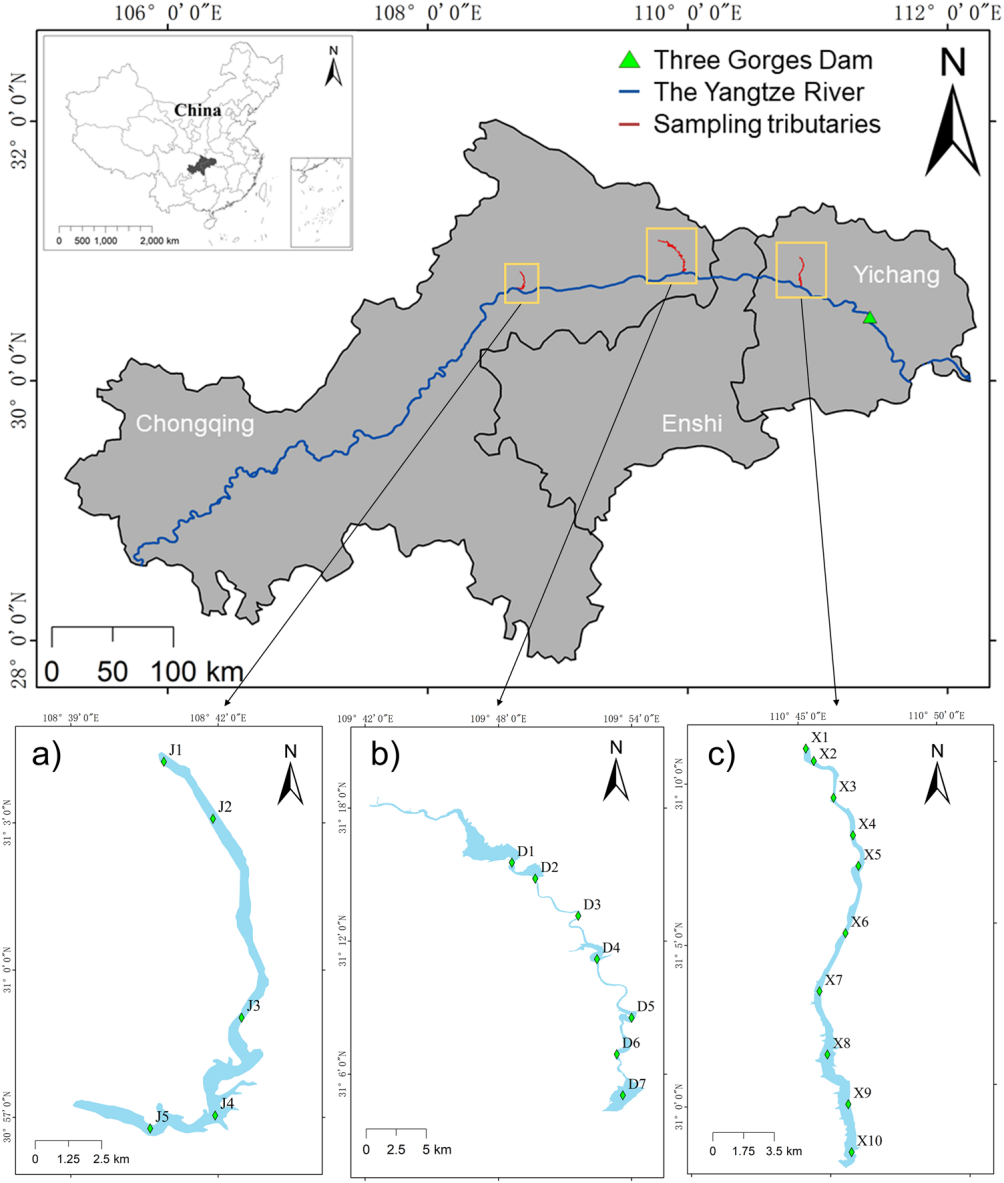
Sampling sites in three typical tributaries of the Yangtze River in TGR area. The three tributaries were (**a**) Xiaojiang River, (**b**) Daning River, and (**c**) Xiangxi River. The basic geographic information was obtained from the National Geomatics Center of China (NGCC) and generated using the ArcGIS 10.7 (http://www.esri.com/).

Xiaojiang River, Daning River, and Xiangxi River are the main tributaries of the Yangtze River in TGR area with a water surface area of 1000 km^2^. The main stream of XiaoJiang River, Daning River, and Xiangxi River is 182.4 km, 250 km, and 97.3 km, respectively. Xiaojiang River basin is mainly surrounded by agricultural land, while Daning River basin and Xiangxi River basin are mainly surrounded by forestry and urban land. According to the length of each sampling tributary, five (J1 − J5), seven (D1 − D7), and ten (X1 − X10) sampling sites were set in Xiaojiang River, Daning River, and Xiangxi River, respectively (Fig. 1). The flow distance between the sampling sites of each tributary was calculated using ArcGIS 10.7. The samples were collected in December 2019. Three parallel sediment samples were collected from a surface of 10 cm at each site, stored in sterile ziplock bags, and taken back to the laboratory on ice. Upon return to the laboratory, the sediments from different sampling sites belonging to same tributary were mixed into one sample, and stored at -80℃ for high-throughput sequencing. The sediment samples were divided into two parts. One part was stored at − 80℃ for DNA extraction, and the other was stored at − 20℃ for physicochemical properties determination.

### Physicochemical analysis

Moisture content of the sediments was measured by weight loss after wet sediment was dried at 105℃ to constant weight. The ammonia nitrogen (NH_4_^+^-N), nitrite nitrogen (NO_2_^–^-N), and nitrate nitrogen (NO_3_^–^-N) were extracted from the sediment samples which were air dried and passed through a 2 mm sieve with 2 mol L^−1^ KCl. The pH of 1:2.5 dry soil / 2 mol L^−1^ KCl (wt/vol) suspensions after 30-min shaking was determined as pH of sediment samples using a pH analyzer (METTLER TOLEDO, Switzerland). Total nitrogen (TN) and total carbon (TC) in sediments were measured using an Element analyzer (Elementar Vario PYRO cube, Germany). Total phosphorus (TP) in sediments was determined by the perchloric acid sulfuric acid method. The physicochemical parameters of all sediment samples were analyzed in triplicate.

### DNA extraction and PCR amplification

The total genomic DNA was extracted from sediment samples using Fast DNA^®^ Spin for Soil Kit (MPBIO, USA) according to the manufacturer’s instructions. The purity and concentration of the extracted DNA samples were determined using a super differential spectrophotometer (NanoPhotometer-N60, IMPLEN, Germany). The specific primer pair including pmoA-189b-F (GGNGACTGGGACTTYTGG) and Com-*amoA*_1_R (CGAGATCATGGTGCTGTGAC) was used to amplify the sediment comammox *Nitrospira amoA* gene^31^. PCR reactions were performed in a total volume of 25μL containing 2μL of template DNA, 2μL of dNTP (2.5 mM, TransGen, China), 5μL of 5 × reaction buffer, 5μL of 5 × GC buffer, 1μL of each primer (10 μM), 8.75μL ddH_2_O, and 0.25μL of Q5^®^ High-Fidelity DNA Polymerase (New England Biolabs, USA). PCRs were conducted as follows: predenaturation at 98 ℃ for 2 min; followed by 30 cycles at 98 ℃ for 15 s, 55 ℃ for 30 s, and 72 ℃ for 30 s; and a final extension at 72 ℃ for 5 min. Agarose gel electrophoresis was conducted to determine the specificity of the amplified products.

### Amplicon sequencing and phylogenetic analysis

PCR products were sequenced using Illumina NovaSeq PE250 by Shanghai Personal Biotechnology Company Limited (Shanghai, China). Raw data were processed using the Vsearch (v2.13.4_linux_x86_64)^32^. The specific treatment process was as follows. First, cutadapt (v2.3) was used to cut the primer fragment and discard the sequences unmatched with primer. Afterwards, paired-end reads were merged and quality filtered using Vsearch. The sequences were clustered to operational taxonomic units (OTUs) according to 97% nucleic acid similarity with chimeras eliminated. Then, singletons OTUs and their representative sequences were removed from the OTU table. Finally, RDP FrameBot (v1.2) was used to correct errors of insertion or deletion in OTU sequences according to the seed protein sequence of comammox *Nitrospira amoA* gene^33^. Neighbor-joining phylogenetic tree of one representative sequence of each main OTU and its closest reference sequence retrieved from GenBank was created using MEGA X with 1000 bootstrap replicates to evaluate the reliability of the tree topologies^34,35^.

### Quantitative real-time PCR (qRT-PCR)

The qRT-PCR was performed to determine the *amoA* gene abundance of comammox *Nitrospira* clade A, comammox *Nitrospira* clade B, AOA, and AOB using QuantStudio™ 6 Flex quantitative PCR instrument (Thermo Fisher Scientific, Singapore). Four primer pairs, namely, CA377f/C576r^36^, CB377f/C576r^36^, Arch-*amoA*F/Arch-*amoA*R^37^, and *amoA*–1Fmod/GenAOBR^38^ were used for the quantification of the aforementioned four *amoA* genes, respectively. Quantitative PCR was carried out in 10 μL amplification system containing 5.0 μL of T5 Fast qPCR Mix (2 ×), 0.4 μL of each Primer (10 μM), 0.2 μL of ROX Reference Dye II (50 ×), 1 μL of template DNA, and 3.0 μL of distilled deionized water (ddH_2_O). The qRT-PCR amplification conditions were shown in Table S1.

### Statistical analysis

The differences in the sediment properties and AOMs *amoA* gene abundance between different groups were tested by one-way ANOVA and Duncan’ test from agricolae package (https://cran.r-project.org/web/packages/agricolae/) and dplyr package (https://dplyr.tidyverse.org) in R v3.6.1^39^ (*P* < 0.05). Standard curve of qPCR process and data generated were analyzed by QuantStudio™ Real-Time PCR Software (version 1.2). Detrended correspondence analysis (DCA) was performed to preliminarily determine environmental parameters. Since the largest value of gradient length obtained from DCA was less than 3.0, redundancy discriminate analysis (RDA) was further performed using vegan package (https://github.com/vegandevs/vegan) in R v3.6.1. Spearman correlation analysis and mapping were conducted using corrplot package (https://github.com/taiyun/corrplot) in R v3.6.1 to test correlations among diversity, abundance and environmental parameters. Heat map of relative abundance of main OTUs was completed using pheatmap package (https://cran.r-project.org/web/packages/pheatmap/) in R v3.6.1. The α-diversity was calculated using QIIME^40^. Co-occurrence network was constructed by the OTUs with relative abundance ≥ 0.02%. Network topological parameters and sparCC coefficients were calculated by igraph package (https://igraph.org) and SpiecEasi package (https://github.com/zdk123/SpiecEasi) in R v3.6.1 and image of co-occurrence network were generated by Gephi 0.9.2 with Fruchterman-Reingold layout^41,42^. Other graphs were prepared using Origin 2018 software. *P* < 0.05 was considered as significantly different.

### Nucleotide sequence accession numbers

The nucleotide sequence of comammox *Nitrospira amoA* gene obtained in this study was submitted to the GenBank database with the accession number of MZ669776 − MZ669807.

### Ethics approval and consent to participate

Not applicable.

### Consent for publication

Not applicable.

## Results

### Environmental parameters

Physical and chemical indexes of sediments from three typical TGR tributaries of the Yangtze River in China were shown in Table 1. The sediments at each sampling point were weakly alkaline (pH > 7). There was no significant difference in moisture content among Xiaojiang River, Daning River, and Xiangxi River. There is little difference in TN content among the three tributaries. However, the NH_4_^+^-N in Xiangxi River was lower than that in other two tributaries with an average of 5.42 mg kg^−1^. Daning River had the highest NO_2_^–^-N with an average of 0.43 mg kg^−1^. The order ranking from high to low in terms of the average NO_3_^–^-N content in three rivers were Xiaojiang River (2.10 mg kg^−1^), Xiangxi River (1.25 mg kg^−1^), and Daning River (0.80 mg kg^−1^). The average TC content in Xiaojiang River was 1.68 g kg^−1^, which was about half as much as that in Daning River (3.26 g kg^−1^). Xiangxi Rievr had the highest average TP content (87.22 mg kg^−1^) among three tributaries.

**Table 1:** Sediment physical and chemical properties for samples collected along the TGR typical tributaries.

Sampling tributaries	Sites	Moisture content (%)	pH	NH_4_^+^-N (mg kg^−1^ dw)	NO_2_^–^-N (mg kg^−1^ dw)	NO_3_^–^-N (mg kg^−1^ dw)	TN (g kg^−1^ dw)	TC (g kg^−1^ dw)	C:N	TP (mg kg^−1^ dw)
Xiaojiang River	J1	48.67 ± 1.95b	7.23 ± 0.06ab	9.07 ± 3.53a	0.27 ± 0.01a	1.03 ± 0.12ab	1.39 ± 0.03b	21.05 ± 0.33a	15.14 ± 0.57a	25.98 ± 15.82b
	J2	60.00 ± 2.73a	7.26 ± 0.20ab	8.20 ± 2.81a	0.16 ± 0.05a	0.56 ± 0.02b	1.44 ± 0.10b	14.84 ± 2.42c	10.31 ± 1.08b	100.59 ± 59.80a
	J3	57.87 ± 2.27a	7.12 ± 0.10b	7.89 ± 1.18a	0.32 ± 0.20a	2.85 ± 0.45a	1.63 ± 0.06a	17.27 ± 0.38b	10.57 ± 0.24b	38.59 ± 0.69b
	J4	37.32 ± 10.43c	7.19 ± 0.04ab	8.28 ± 1.24a	0.14 ± 0.08a	1.18 ± 0.45ab	1.17 ± 0.04c	9.01 ± 0.86d	7.72 ± 0.99c	81.84 ± 20.86ab
	J5	50.60 ± 2.20b	7.41 ± 0.03a	5.16 ± 1.67a	0.26 ± 0.06a	0.61 ± 0.05b	1.42 ± 0.06b	22.06 ± 0.49a	15.54 ± 0.78a	58.60 ± 10.93ab
Daning River	D1	41.36 ± 4.73c	7.48 ± 0.10a	6.27 ± 1.55b	0.39 ± 0.11a	1.53 ± 0.97a	1.04 ± 0.13d	55.25 ± 3.70a	53.13 ± 10.01a	41.03 ± 8.07bc
	D2	57.42 ± 5.13ab	7.42 ± 0.07a	5.99 ± 1.92b	0.48 ± 0.08a	0.34 ± 0.09b	1.49 ± 0.09b	34.84 ± 1.43b	23.38 ± 1.99bc	55.40 ± 2.96abc
	D3	59.96 ± 4.20a	7.42 ± 0.09a	12.64 ± 3.54a	0.57 ± 0.09a	0.78 ± 0.14ab	1.73 ± 0.03a	27.53 ± 0.23c	15.91 ± 0.30 cd	55.82 ± 25.47abc
	D4	53.06 ± 2.87b	7.42 ± 0.12a	7.04 ± 1.61ab	0.15 ± 0.01a	0.67 ± 0.17ab	1.20 ± 0.24 cd	28.26 ± 1.18c	23.55 ± 4.25b	64.29 ± 6.34ab
	D5	55.32 ± 2.44ab	7.15 ± 0.22b	10.40 ± 4.18ab	0.38 ± 0.32a	1.61 ± 0.54a	1.32 ± 0.08bc	34.45 ± 0.02b	26.10 ± 1.54b	39.04 ± 11.37bc
	D6	56.03 ± 3.39ab	7.49 ± 0.12a	5.64 ± 0.33b	0.16 ± 0.03a	0.38 ± 0.19b	1.44 ± 0.05b	26.85 ± 3.36c	18.69 ± 1.98bcd	29.57 ± 11.57c
	D7	55.71 ± 1.15ab	7.43 ± 0.04a	5.93 ± 1.64b	0.26 ± 0.05a	0.32 ± 0.11b	1.53 ± 0.05ab	20.93 ± 0.50d	13.68 ± 0.52d	80.02 ± 25.98a
Xiangxi River	X1	52.92 ± 4.15b	7.38 ± 0.04a	7.28 ± 1.33a	0.97 ± 0.01a	0.73 ± 0.01ab	1.48 ± 0.11 cd	39.61 ± 0.94a	26.83 ± 2.57a	83.25 ± 32.49a
	X2	44.00 ± 6.29c	7.41 ± 0.27a	9.35 ± 3.88a	0.75 ± 0.34ab	3.51 ± 2.98ab	1.46 ± 0.05 cd	36.00 ± 0.67ab	24.66 ± 1.30a	93.20 ± 29.32a
	X3	53.93 ± 0.95b	7.27 ± 0.17a	4.35 ± 1.03ab	0.19 ± 0.10c	2.38 ± 1.89ab	1.70 ± 0.04a	30.91 ± 0.43bc	18.18 ± 0.70bc	75.32 ± 44.24a
	X4	54.02 ± 6.90b	7.55 ± 0.08a	0.40 ± 0.03b	0.05 ± 0.00c	3.58 ± 0.14ab	1.39 ± 0.02d	19.06 ± 3.62ef	13.68 ± 2.68cde	105.15 ± 38.10a
	X5	51.58 ± 0.80b	7.31 ± 0.08a	6.94 ± 2.45ab	0.18 ± 0.12c	0.51 ± 0.19b	1.46 ± 0.07 cd	27.57 ± 0.55 cd	18.93 ± 0.93b	121.30 ± 26.93a
	X6	58.62 ± 2.73a	7.29 ± 0.03a	9.68 ± 2.42a	0.06 ± 0.00c	3.18 ± 1.62ab	1.65 ± 0.12ab	19.02 ± 4.44ef	11.51 ± 2.22de	80.59 ± 29.43a
	X7	54.72 ± 0.51ab	7.49 ± 0.03a	6.74 ± 3.19ab	0.05 ± 0.01c	1.80 ± 1.40ab	1.56 ± 0.03bc	26.18 ± 11.99cde	16.78 ± 7.44bcd	76.84 ± 8.36a
	X8	55.08 ± 1.18ab	7.53 ± 0.06a	5.56 ± 0.67ab	0.46 ± 0.12bc	0.52 ± 0.11b	1.49 ± 0.09 cd	16.42 ± 0.88f.	11.02 ± 0.32e	60.61 ± 24.27a
	X9	52.61 ± 1.72b	7.32 ± 0.19a	3.59 ± 0.17ab	0.09 ± 0.02c	0.28 ± 0.10b	1.41 ± 0.07d	16.74 ± 0.64f.	11.90 ± 0.27de	113.09 ± 22.26a
	X10	53.65 ± 2.39b	7.53 ± 0.00a	0.37 ± 0.00b	0.08 ± 0.03c	4.47 ± 0.04a	1.51 ± 0.10 cd	21.60 ± 0.02def	14.34 ± 0.95bcde	62.90 ± 13.28a

Different lower-case letters indicate a significant difference among different sites in the same tributary at the level of *P* < 0.05 by Duncan’ test.

### Abundance of Comammox *Nitrospira amoA* gene and Canonical Ammonia Oxidizers *amoA* gene

**Figure 2:**
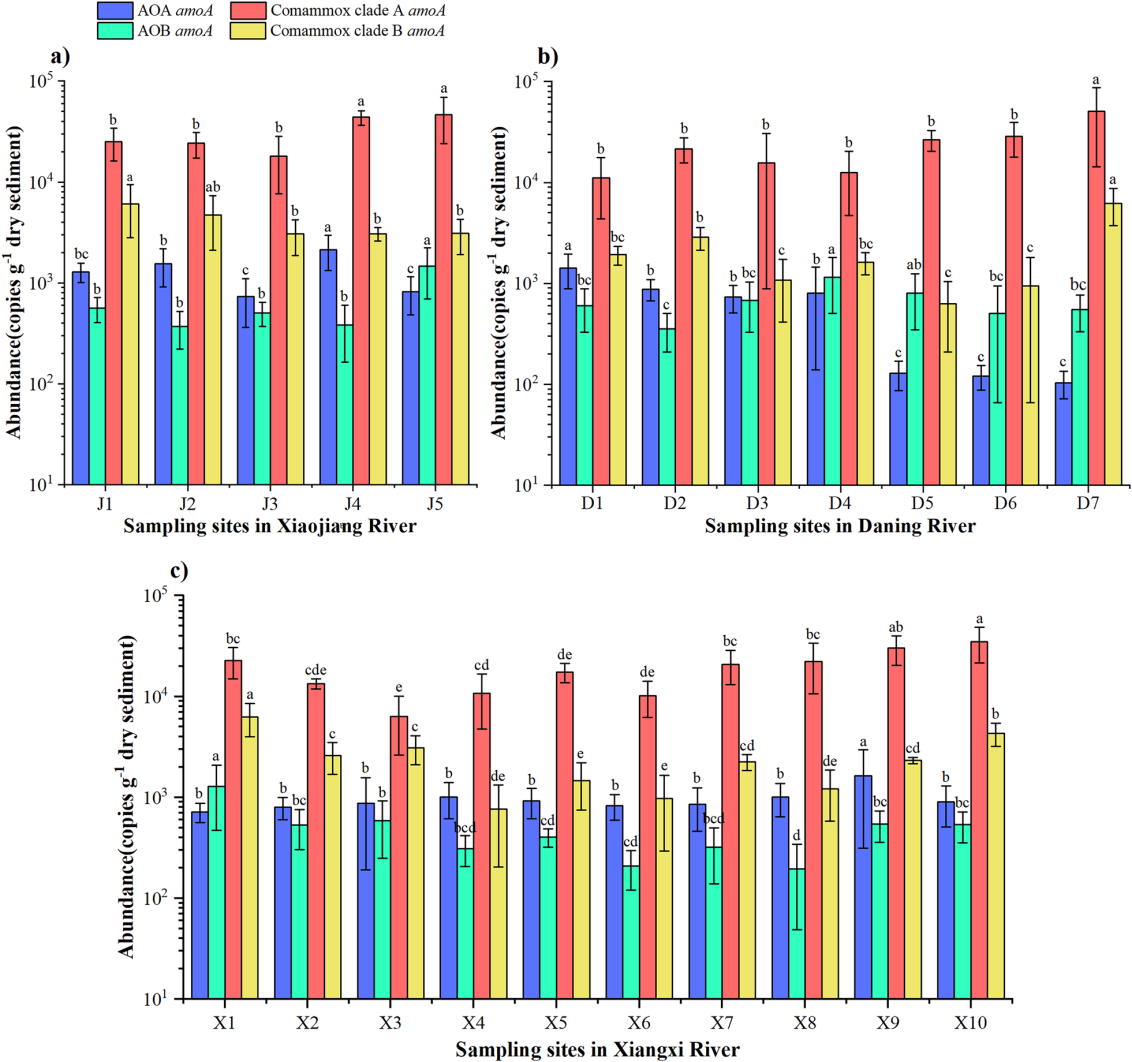
The *amoA* gene abundance distribution of AOA, AOB, Comammox *Nitrospira* clade A, and Comammox *Nitrospira* clade B in (**a**) Xiaojiang River, (**b**) Daning River, and (**c**) Xiangxi River. Different lower-case letters indicate a significant difference among different AOMs at the level of *P* < 0.05 by Duncan’ test.

In the sediments from three tributaries in TGR area, the comammox *Nitrospira* clade A *amoA* gene showed a gradual increasing abundance along the flow path (Fig. 2). The clade A abundance reached the maximum at the estuary of the three tributaries. Similar to the spatial variation of clade A abundance, clade B *amoA* gene abundance was also increased gradually with the flow path, and its abundance reached the highest at the estuary of Daning River. However, the clade B *amoA* gene abundance in the other two rivers showed an overall decreasing trend along the flow path.

**Figure 3:**
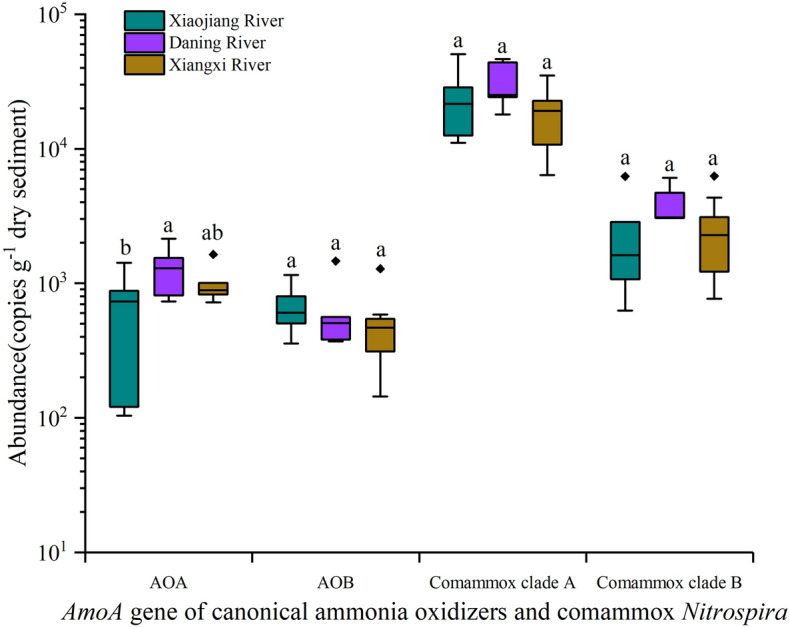
*AmoA* gene abundance of comammox *Nitrospira* and canonical AOMs in the sediments of sampling tributaries. Different lower-case letters indicate a significant difference among different AOMs at the level of *P* < 0.05 by Duncan’ test.

In the total of 22 samples from three TGR tributaries, the *amoA* gene abundance of comammox *Nitrospira* clade A (6.36 × 10^3^ − 5.06 × 10^4^ copies g^−1^ dry sediment) was significantly higher than that of comammox *Nitrospira* clade B (6.26 × 10^2^ − 6.27 × 10^3^ copies g^−1^ dry sediment), AOA (1.03 × 10^2^ − 2.14 × 10^3^ copies g^−1^ dry sediment), and AOB (1.44 × 10^2^ − 1.46 × 10^3^ copies g^−1^ dry sediment) (Fig. 3, *P* < 0.05). Moreover, the *amoA* gene abundance of comammox *Nitrospira* clade B was also significantly higher than that of AOA and AOB in the three tributaries (Fig. 3, *P* < 0.05). In addition, AOA *amoA* gene abundance was significantly higher than AOB *amoA* gene abundance in Xiaojiang River and Xiangxi River, but there was no significant difference in *amoA* gene abundance between these two microorganisms in Daning River. In AOMs, AOA was the only one exhibiting significant difference in *amoA* gene abundance among tributaries. AOA *amoA* gene abundance in Daning River was significantly higher than that in Xiaojiang River (Fig. 3, *P* < 0.05, Duncan’ test).

### Effect of physicochemical characteristics of sediments on *amoA* gene abundance of AOMs

**Figure 4:**
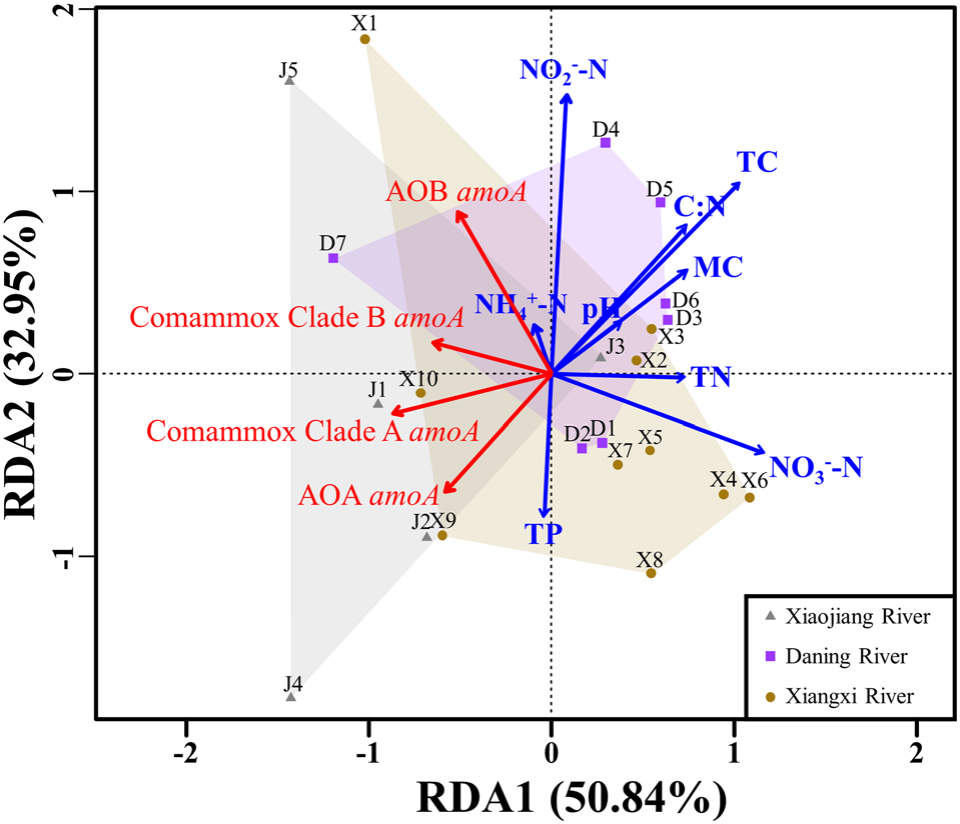
RDA plots of the environmental factors and *amoA* gene abundance of AOMs in three tributaries.

**Figure 5:**
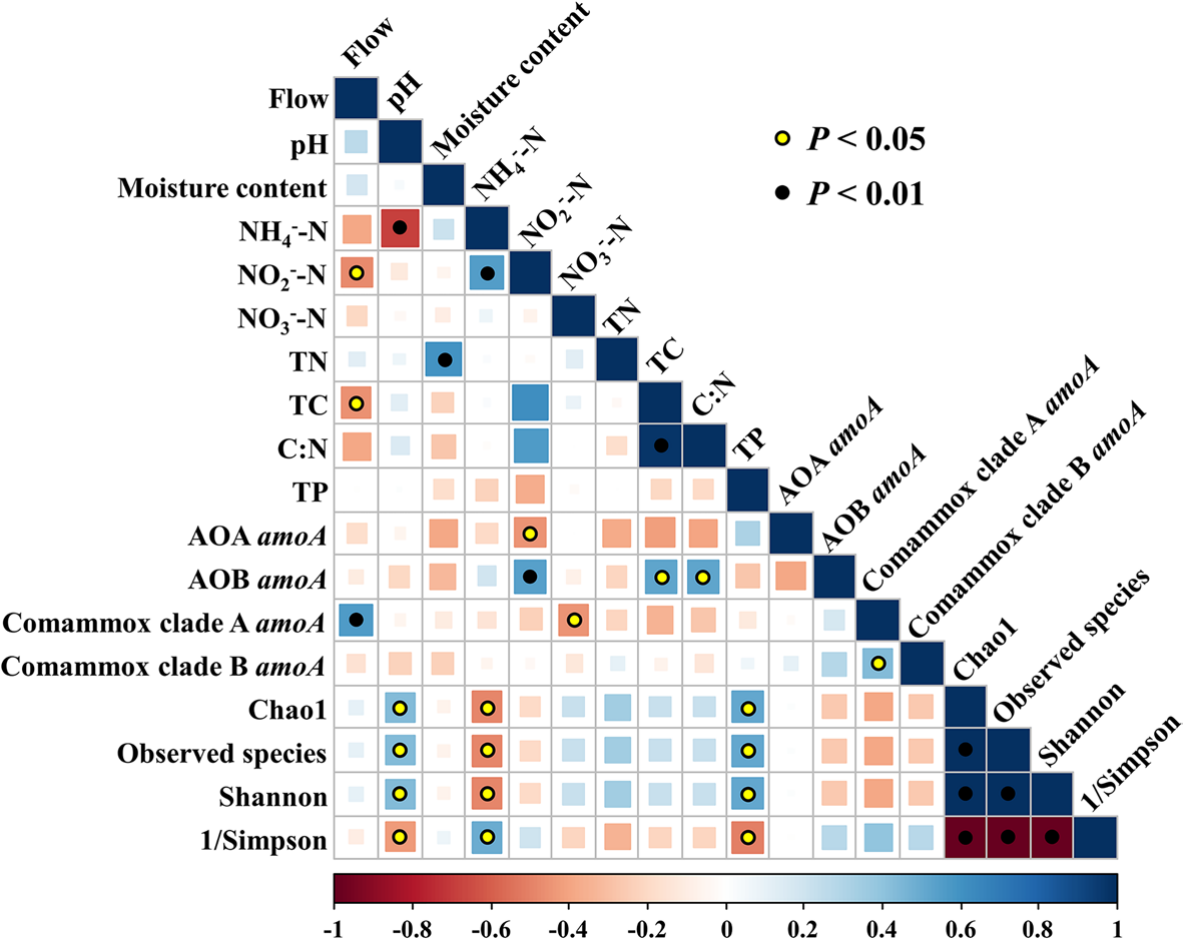
Heatmap exhibits the pairwise Spearman’s correlations among environmental parameters, *amoA* gene abundance of AOMs, and α-diversity indices of comammox *Nitrospira* community. Only the significant correlations (*P* < 0.05) are labeled in circles. Different colors of scale bars (at the bottom) indicate different correlation coefficient values.

The first two RDA axes in three tributaries’ RDA diagram jointly explained 83.8% of species-environment relationships (Fig. 4). Comammox *Nitrospira* clade A *amoA* gene abundance was significantly positively correlated with flow distance (Fig. 5, r = 0.564, *P* < 0.01, n = 22). *AmoA* gene abundance showed a significant positive correlation between the two lineages of comammox *Nitrospira* (Fig. 5, r = 0.433, *P* < 0.05, n = 22), and a significant positive correlation in *amoA* gene abundance was also observed between comammox *Nitrospira* clade A and AOA (Fig. 4).

In addition, the *amoA* gene abundance of AOA and AOB was positively correlated with sediment TP and NH_4_^+^-N, respectively (Fig. 4). AOB *amoA* gene abundance was also significantly positively correlated with sediment NO_2_^–^-N content (r = 0.542, *P* < 0.01, n = 22), TC content (r = 0.517, *P* < 0.05, n = 22), and C:N ratio (r = 0.523, *P* < 0.05, n = 22) (Fig. 5).

### Biodiversity and community of comammox *Nitrospira*

All the generated 191692 high-quality comammox *Nitrospira amoA* gene sequences were clustered into 5086 OTUs based on 97% nucleotide similarity. The α-diversity for each point was shown in Table S2. Spearman correlation analysis indicated that sediment pH, NH_4_^+^-N, and TP were significantly correlated with all the α-diversity indices of comammox *Nitrospira* community (*P* < 0.05, Fig. 5).

**Figure 6:**
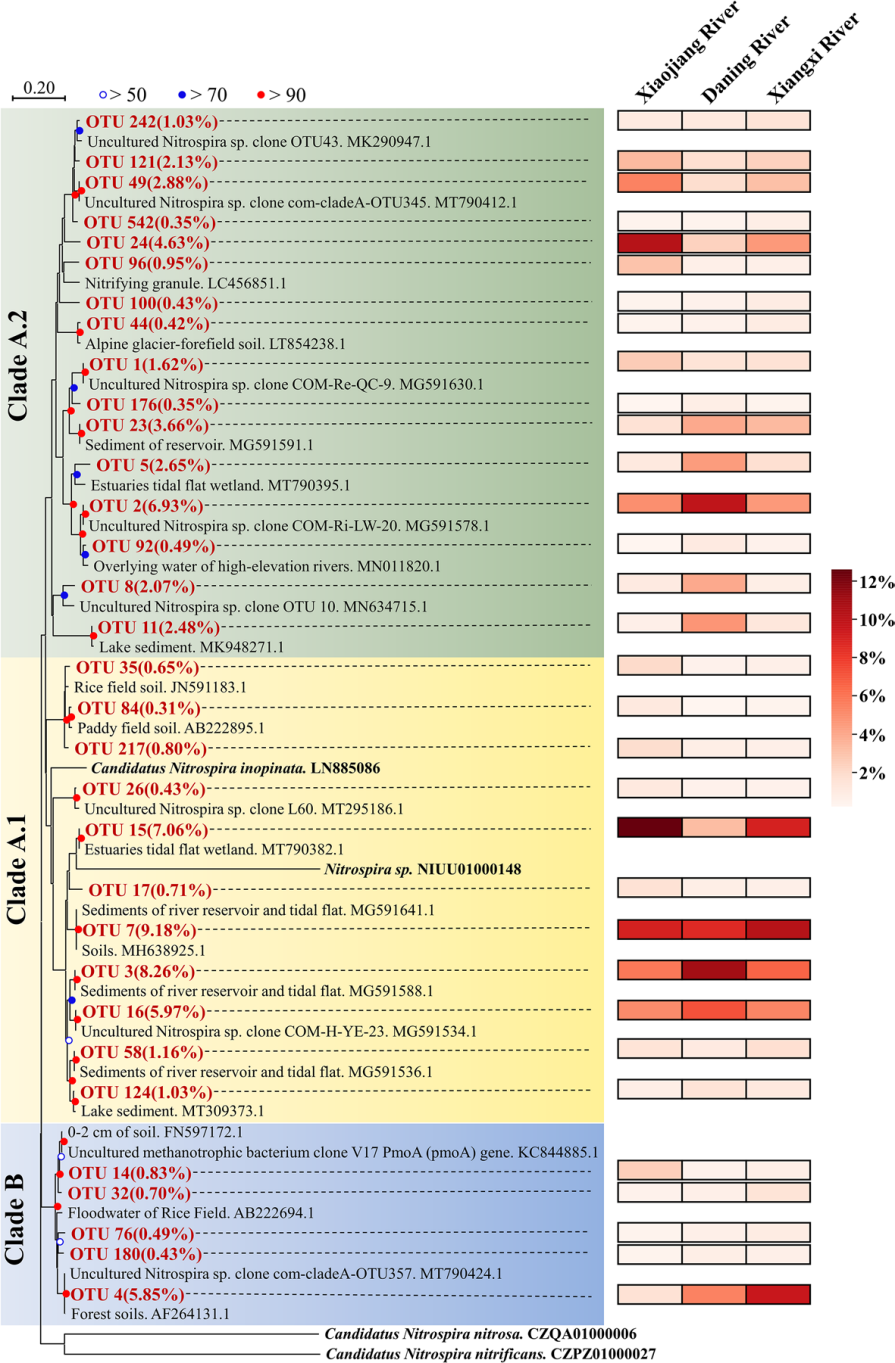
Neighbor-joining phylogenetic tree and relative abundances of Comammox *Nitrospira amoA* gene sequences. The reference sequences are from the GenBank. The sequence and its corresponding sequence number (consisting of the letters and numbers, behind sequence) are listed in a row. Percentages in brackets following the OTUs indicate the percentage of each OTU in the total comammox *amoA* gene sequences. The calculation method of neighbor-joining phylogenetic tree is Maximum Composite Likelihood, and the scale bar (on the top) represents 20% sequence divergence.

**Figure 7:**
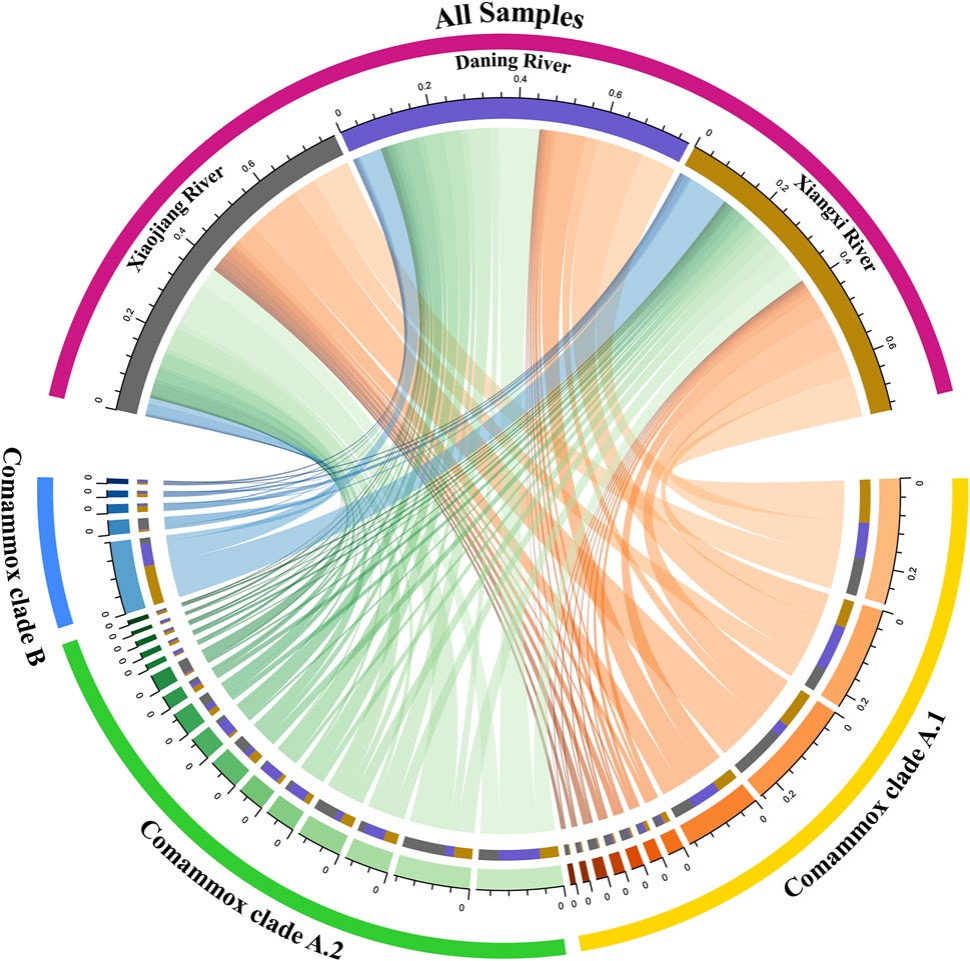
Distribution of 32 main OTUs among different sampling tributaries.

**Figure 8:**
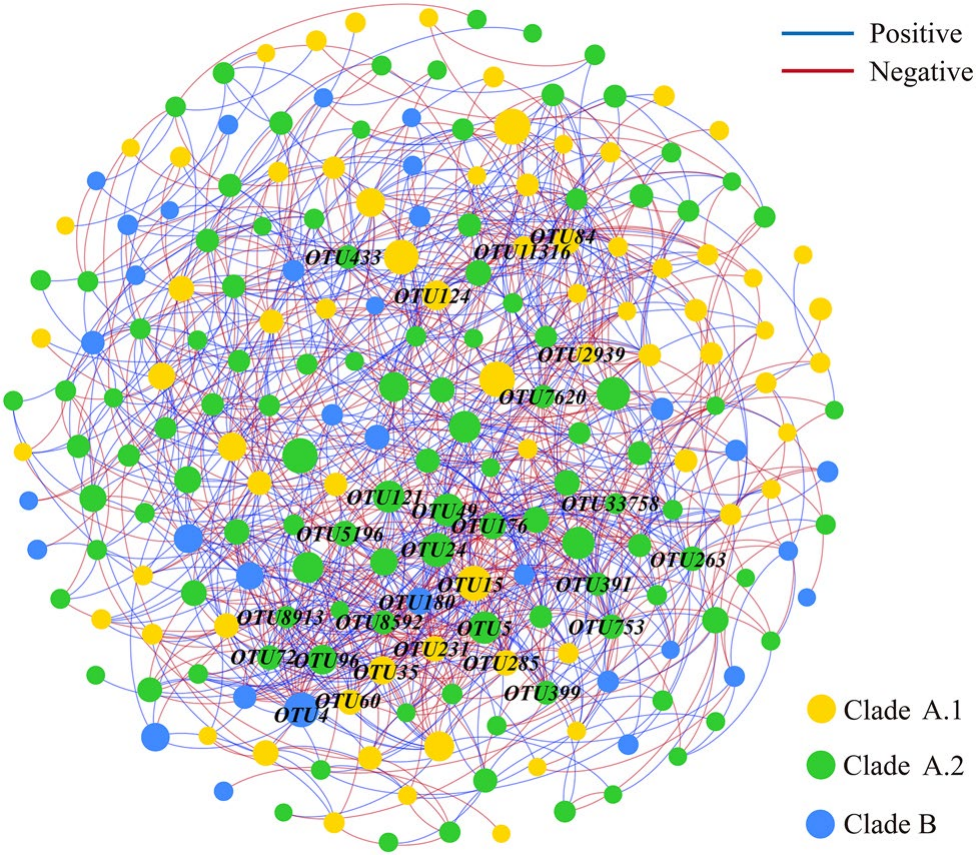
Co-occurrence network analysis of comammox *Nitrospira*. The nodes represented OTUs, the relationship between two OTUs in the network was shown by an edge. The inferred correlations were restricted to those having correlations > 0.3 or < − 0.3 (*P* < 0.05, two-sided). Nodes with different colors represent different clades of comammox *Nitrospira*, and the size of the nodes was proportional to their relative abundance. The bule and red edges represent the positive and negative correlations, respectively, and the thickness of each edge between two nodes is proportional to the value of correlation coefficient. Hub nodes (degree > 20) were labeled in network.

The phylogenetic analysis revealed that the 32 main OTUs with a relative abundance > 0.3% accounted for 76.9% of the total comammox *Nitrospira amoA* gene sequences (Fig. 6). The phylogenetic tree supported the division of the 32 main OTUs of comammox *Nitrospira amoA* gene into comammox *Nitrospira* into clade A (27 OTUs) and clade B (5 OTUs). Clade A was further subdivided into clade A.1 (11 OTUs) and A.2 (16 OTUs). Clade A.1, clade A.2, and clade B were found in the sediments from all three tributaries. Clade A.1 accounted for 40.6% and 35.2% of comammox *Nitrospira* community in Xiaojiang River and Xiangxi River, and it was the most dominant lineage in these two tributaries. Clade A.2 was the most dominant lineage in Daning River, accounting for 38.0% of comammox *Nitrospira* community (Fig. 7). Clade B was not the dominant lineage in all three tributaries with its highest percentage found in Xiangxi River (12.4%). The relationship of 214 OTUs with relative abundance > 0.02% was explored with co-occurrence network analysis (Fig. 8). The connections among the members in network were mostly positive, accounting for 54.0% (Table S3). It was noteworthy that nodes of clade A.1, cladeA.2, and clade B accounted for 33.2%, 51.4%, and 15.4% of all nodes, respectively, and 9, 17, and 2 of 28 hub nodes, respectively. In addition, the average degrees of clade A.1, clade A.2 and clade B were 10.4, 12.0 and 8.1, respectively (Table S4). Clade A.2 not only had the most OTUs and the highest relative abundance in phylogenetic analysis, but also occupied the most nodes and had the highest average degree in network analysis.

## Discussion

### Ecological differentiation of two lineages of comammox *Nitrospira*

In the TGR area of the Yangtze River in China, the *amoA* gene abundance of comammox *Nitrospira* clade A in three tributaries and clade B in one tributary was gradually increased along the flow path, but *amoA* gene abundance of clade B was gradually decreased in two tributaries, indicating that the spatial change of tributaries led to the niche change of comammox *Nitrospira*. One previous study found that comammox *Nitrospira* had niche differentiation along the flow path in the main stream of the Yangtze River^30^. Similarly, the niche differentiation of comammox *Nitrospira* have also been reported in the estuary ecosystem^19^.

Landforms and nutrient conditions have been assumed to lead to the differentiation of comammox *Nitrospira* in the main stream of the Yangtze River^30^. However, local ammonium concentration might play the main role in the niche differentiation between comammox *Nitrospira* and canonical AOMs in agricultural ecosystem^43^. In this study, the abundance of clade A showed a significant positive correlation with the flow path (*P* < 0.05, Fig. 5), indicating that the spatial change of hydrology and water quality of tributaries might be the main driving force for niche differentiation of this microorganism. In the TGR, the closer the tributary is to the estuary, the slower the flow velocity is since the main stream of Yangtze River has a supporting effect on the tributary. Fine sediments are easy to deposit in the area close to the estuary, resulting in spatial differences in tributary sediments along the flow. In addition, the tributary gradually mixes with the main stream in the process of flowing downstream, thus leading to spatial difference in the water bodies of tributaries along the flow path. These two spatial differences might be the main reason for the niche differentiation of comammox *Nitrospira* along the flow path in this study.

Among the three tributaries in the TGR area, comammox *Nitrospira* community also exhibited ecological differentiation. Clade A was the dominant lineage in all three tributaries, of which clade A.1 was the dominant lineage in Xiaojiang River and Xiangxi River, and clade A.2 was the dominant lineage in Daning River. Clade B was dominant in none of the three tributaries. These results showed that different comammox *Nitrospira* lineages were adapted to the water environment of different tributaries, resulting in niche differentiation. One study found that soil pH was mainly responsible for the differentiation of comammox *Nitrospira* lineage^44^, while another study reported that low nutrient environment was the main reason for the differentiation of comammox *Nitrospira* lineage^45^. In this study, the difference in hydrological regimes and sediment nutrient conditions might lead to the dominance of clade A.1 and clade A.2 in different tributaries. However, the more specific reasons remain to be further explored in combination with the tributary environment and the physiological characteristics of the two sub-clades.

### Coexistence and niche differentiation of Comammox *Nitrospira* and Canonical AOMs

This study found the coexistence of comammox *Nitrospira* with AOA and AOB in all sampling sites. Many studies have also revealed that AOMs could coexist in forest soil, agricultural soil, lake sediment, and other environments^46–48^. In this study, *amoA* gene abundance of comammox *Nitrospira* clade A was the highest, followed by clade B. The *amoA* gene abundance of comammox *Nitrospira* at each sampling site was about one order of magnitude higher than that of canonical AOMs, indicating that comammox *Nitrospira* might play a major role in the ammonia oxidation process in the sediment of TGR’s tributaries. Comammox *Nitrospira* have been reported to be dominant in forest, grassland, and agricultural soil environments^49–51^.

The niche differentiation of AOA and AOB in soil environment was mainly determined by ammonia limitation, pH, and mixotrophy^2^. AOA and AOB had competitive advantages respectively in acidic and high-ammonium concentration environments^52^. Kinetic and genomic studies showed that comammox *Nitrospira* was more likely to grow in microaerobic and oligotrophic environments^23,24^. Moreover, comammox *Nitrospira* also exhibited a preference for slightly alkaline environments^53^. In this study, the α-diversity indices of comammox *Nitrospira* community (either Chao1 index and Observed Species index representing species richness, or Shannon index and 1/Simpson index representing diversity) showed a significant negative correlation with sediment NH_4_^+^-N content (*P* < 0.05, Fig. 5). These evidences showed that comammox *Nitrospira* were more likely to grow in the ammonium limitation area in the TGR. In addition, niche differentiation among these AOMs led to subsequent environmental effects. The generation of nitrite in two-step nitrification could be avoided in one-step nitrification process, comammox *Nitrospira*, thus reducing nitrite concentration in the environment. Comammox *Nitrospira* genomes contain no enzymes related to nitrogen oxide metabolism such as cytochrome c nitric oxide reductase (cNOR), thereby decreasing the risk of N_2_O production^7^. Kits et al. (2019) showed that the contribution of *Ca. Nitrospira inopinata* to N_2_O emission was similar to that of AOA, but much lower than that of AOB^54^. Han et al. (2021) also reported that the N_2_O and NO_y_ production capacity of comammox *Nitrospira* was only 3 − 15% of AOA and AOB^55^. More attention should be paid to the subsequent environmental effects induced by these niche differentiations.

### Community structure of comammox *Nitrospira*

The main OTUs in this study exhibited high similarity with the sequences of comammox *Nitrospira* existing in a variety of freshwater and terrestrial ecosystems (Fig. 6). The sequences of OTU 3, OTU 7, OTU 16, OTU 26, and OTU 58 in clade A.1 were extremely similar to the sequences of comammox *Nitrospira* in the sediments of river, reservoir and tidal flat^56^. In addition, OTU 7 and OTU 124 in the same lineage (clade A.1) had high homology with the sequences of comammox *Nitrospira* from wetland soil of Qinghai-Tibetan plateau and lake sediment, respectively^9,48^. The sequences of OTU 49, OTU 44, and OTU 11 in clade A.2 had high homology with the sequences of comammox *Nitrospira* from estuary tidal flat wetland, alpine glacier-forefield soil and lake sediment, respectively^10^. The sequences of OTU 4 in clade B had high similarity with the sequences of comammox *Nitrospira* from forest soils (> 95%, 1000 replicates)^57^.

Phylogenetic analysis also showed that comammox *Nitrospira* in the sediments of the TGR tributaries had a complex community structure. In all the samples, Clade A and clade B coexisted, and clade A.1 and clade A.2 also coexisted. The coexistence of clade A and clade B has also been reported in tidal flat wetland and agricultural soil^12,45^. However, only clade A was found in tidal sediments, plateau wetland and estuarine sediments^56,58,59^. Our data showed the coexistence of clade A.1, clade A.2, and clade B in the sediment from the same tributary in the TGR area, indicating that the TGR was suitable for the survival of comammox *Nitrospira*. Co-occurrence network analysis showed that clade A.2 was critical to comammox *Nitrospira* community, which was similar to the conclusion reported in plateau wetland sediments and forest soils^44,59^. Comammox *Nitrospira* may play an important role in the nitrogen cycle of the TGR. However, its ammonia oxidation effect needs to be further studied.

## Conclusions

This study revealed that comammox *Nitrospira* widely existed in tributary sediments of the TGR, meanwhile, clade A and clade B existed simultaneously. The *amoA* gene abundance of clade A and clade B exhibited a spatial change trend of gradual increase or decrease along the flow path. The *amoA* abundance of comammox *Nitrospira* clade A in the sediments showed a significant positive correlation with the flow distance between sampling sites. The two sub-clades of clade A were dominant in different tributaries, indicating that clade A had niche differentiation among different tributaries. These findings confirmed that niche differentiation of comammox *Nitrospira* community appeared in the tributary of the TGR and clade A.2 played an important role in comammox *Nitrospira* community.

## Supplementary Information

Supplementary Information.

## Data Availability

The nucleotide sequence of comammox *Nitrospira amoA* gene obtained in this study was submitted to the GenBank database with the Accession Number of MZ669776 – MZ669807.
