# Supplementary material for: Niche differentiation of comammox Nitrospira in sediments of the Three Gorges Reservoir typical tributaries, China
Source: Sci Rep. 2022 Apr 26;12:6820. doi: 10.1038/s41598-022-10948-9 (PMC9042867; doi:10.1038/s41598-022-10948-9)
Supplement: Supplementary file 1 — Supplementary Information. [file 41598_2022_10948_MOESM1_ESM.pdf]

Niche Differentiation of Comammox *Nitrospira* in sediments of the Three Gorges Reservoir typical tributaries, China

Jiahui Zhang [a, b, c], Mingming Hu [a, b, *], Yuchun Wang [a, b], Jianwei Zhao [c, *], Shanze Li [a, b], Yufei Bao [a, b], Jie Wen [a, b], Jinlong Hu [c], Mingzhi Zhou [c]

a State Key Laboratory of Simulation and Regulation of Water Cycle in River Basin, Beijing 10038, China.

b Department of Water Ecology and Environment, China Institute of Water Resources and Hydropower Research, Beijing 100038, China

c Laboratory of Eco-Environmental Engineering Research, State Environmental Protection Key Laboratory of Soil Health and Green Remediation, College of Resources and Environment, Huazhong Agricultural University, Wuhan 430070, China

* Mingming Hu and Jianwei Zhao are Co-corresponding authors.

Mingming Hu: Tel: +86-10-68781761; E-mail: hmmkeke@163.com; Institute: China Institute of Water Resources and Hydropower Research; Post address: Yangfangdian Road, Haidian District, Beijing, People's Republic of China; Postcode: 100038

Jianwei Zhao: Tel: +86-27-85669068; E-mail: jwzhao2@163.com; Institute: College of Resources and Environment, Huazhong Agricultural University; Post address: No.1, Shizishan Street, Hongshan District, Wuhan City, Hubei Province, People's Republic of China; Postcode: 430070

Table S1. qPCR primers and amplificaiton protocols in this study

Target genes	Primers	Primer sequences (5′-3′)	Reference	qPCR conditions
Comammox *amoA* clade A	CA377f	GTGGTGGTGGTCBAAYTA	(Jiang et al., 2020) [1]	95 °C for 1 min; 40 cycles of 10 s at 95 °C, 20 s at 52 °C, 30 s at 72 °C.
	C576r	GAAGCCCATRTARTCNGCC		
Comammox *amoA* clade B	CB377f	GTACTGGTGGGCBAAYTT	(Jiang et al., 2020) [1]	95 °C for 1 min; 40 cycles of 10 s at 95 °C, 20 s at 52 °C, 30 s at 72 °C.
	C576r	GAAGCCCATRTARTCNGCC		
AOA *amoA*	Arch-amoAF	STAATGGTCTGGCTTAGACG	(Francis et al., 2005) [2]	95 °C for 1 min; 40 cycles of 10 s at 95 °C, 20 s at 56 °C, 30 s at 72 °C.
	Arch-amoAR	GCGGCCATCCATCTGTATGT		
AOB *amoA*	amoA-1Fmod	CTGGGGTTTCTACTGGTGGTC	(Meinhardt et al., 2015) [3]	95 °C for 1 min; 40 cycles of 10 s at 95 °C, 20 s at 52 °C, 30 s at 72 °C.
	GenAOBR	GCAGTGATCATCCAGTTGCG		

Table S2. The α-diversity indices of comammox bacteria

Sampling tributaries	Chao1	Shannon	1 / Simpson	Observed species
Xiaojiang River	1776.81	5.30	1.07	680
Daning River	2301.87	5.82	1.05	913
Xiangxi River	2761.88	6.20	1.05	1072

Table S3. The numbers of positive and negative connections in network of typical tributary sediments from TGR

Connections	Connections **within** each clade	Connections **between** each clade	Total connections
Positive	320 (27.5%)	309 (26.5%)	629 (54.0%)
Negative	219 (18.8%)	317 (27.2%)	536 (46.0%)
Total connections	539 (46.3%)	626 (53.7%)	1165

Table S4. The number of nodes and node features in network of typical tributary sediments from TGR

Node features	Clade A.1	Clade A.2	Clade B	Total
Nodes	71 (33.2%)	110 (51.4%)	33 (15.4%)	214
Average degree	10.5	12.0	8.1	10.9
Hub nodes (Degree > 20)	9 (32.1%)	17 (60.7%)	2 (7.1%)	28

Reference

1. Jiang, R. *et al.* Use of Newly Designed Primers for Quantification of Complete Ammonia-Oxidizing (Comammox) Bacterial Clades and Strict Nitrite Oxidizers in the Genus *Nitrospira*. *Appl. Environ.*

Microbiol. **86**(20), e01775-20. https://doi.org/10.1128/AEM.01775-20 (2020).

2. Francis, C. A., Roberts, K. J., Beman, J. M., Santoro, A. E. & Oakley, B. B. Ubiquity and diversity of ammonia-oxidizing archaea in water columns and sediments of the ocean. *Proc. Natl. Acad. Sci.* **102**(41), 14683–14688 (2005).
3. Meinhardt, K. A. *et al.* Evaluation of revised polymerase chain reaction primers for more inclusive quantification of ammonia-oxidizing archaea and bacteria. *Environ. Microbiol. Rep.* **7**(2), 354–363 (2015).
